# Potential Benefits of N-Acetylcysteine in Preventing Pregabalin-Induced Seeking-Like Behavior

**DOI:** 10.3390/healthcare9040376

**Published:** 2021-03-29

**Authors:** Atiah H. Almalki, Hashem O. Alsaab, Walaa F. Alsanie, Ahmed Gaber, Turki Alkhalifa, Ahmad Almalki, Omar Alzahrani, Ana Maria Gregio Hardy, Qasim Alhadidi, Zahoor A. Shah, Yusuf S. Althobaiti

**Affiliations:** 1 Department of Pharmaceutical Chemistry, College of Pharmacy, Taif University, P.O. Box 11099, Taif 21944, Saudi Arabia; ahalmalki@tu.edu.sa; 2 Addiction and Neuroscience Research Unit, Taif University, P.O. Box 11099, Taif 21944, Saudi Arabia; h.alsaab@tu.edu.sa (H.O.A.); w.alsanie@tu.edu.sa (W.F.A.); a.gaber@tu.edu.sa (A.G.); O07mar@hotmail.com (O.A.); 3 Department of Pharmaceutics and Pharmaceutical Technology, College of Pharmacy, Taif University, P.O. Box 11099, Taif 21944, Saudi Arabia; 4 Department of Clinical Laboratories Sciences, The Faculty of Applied Medical Sciences, Taif University, P.O. Box 11099, Taif 21944, Saudi Arabia; 5 Department of Biology, College of Science, Taif University, P.O. Box 11099, Taif 21944, Saudi Arabia; 6 Ministry of Interior, General Directorate of Narcotics Control, General Administration for Precursors and Laboratories, Riyadh 11564, Saudi Arabia; Turki2004_25@hotmail.com (T.A.); Ah1180mad@hotmail.com (A.A.); 7 Department of Physiology and Pharmacology, College of Medicine and Life Sciences, University of Toledo, Toledo, OH 43614, USA; AnaMaria.Hardy@UToledo.Edu; 8 Department of Anesthesiology, Perioperative and Pain Medicine, Stanford Medical School, Stanford University, Stanford, CA 94305, USA; qalhad18@stanford.edu; 9 Department of Medicinal and Biological Chemistry, College of Pharmacy and Pharmaceutical Sciences, University of Toledo, Toledo, OH 43614, USA; zahoor.shah@utoledo.edu; 10 Department of Pharmacology and Toxicology, College of Pharmacy, Taif University, P.O. Box 11099, Taif 21944, Saudi Arabia

**Keywords:** NAC, pregabalin, addiction, GLT-1, xCT, CPP

## Abstract

Substance-use disorder is globally prevalent and responsible for numerous social and medical problems. Pregabalin (Lyrica), typically used to treat diabetic neuropathy, has recently emerged as a drug of abuse. Drug abuse is associated with several neuronal changes, including the downregulation of glutamate transporters such as glutamate transporter 1 and cystine/glutamate antiporter. We investigated the effects of N-acetylcysteine, a glutamate transporter 1 and xCT upregulator, on pregabalin addiction using a conditioned place preference paradigm. Pregabalin (60 mg/kg) was found to induce conditioned place preference when compared to a vehicle. A 100 mg/kg dose of N-acetylcysteine was found to block pregabalin-seeking behaviors. These results support previous findings showing that glutamate transporters play an important role in pregabalin-induced seeking behaviors. N-acetylcysteine may represent a beneficial agent in preventing the abuse potential of pregabalin.

## 1. Introduction

Pregabalin, a gamma-aminobutyric acid derivative, reduces the release of glutamate by binding to a voltage-gated calcium channel subunit [1]. Pregabalin is predominantly used to treat neuropathy in diabetic patients, anxiety disorder, and partial epilepsy [2,3]. Recently, pregabalin emerged as an illicit drug widely abused throughout the world [4,5,6], especially among Saudi Arabian youth [7,8]. This may be due to the lack of regulations and knowledge about pregabalin’s addictive properties. High doses and extended use of pregabalin were reported to be associated with euphoria and withdrawal symptoms [9,10,11,12]. Althobaiti et al. showed that pregabalin induced conditioned place preference (CPP) and might affect the glutamatergic mechanism when used at doses of 60–90 mg/kg [13]. It is essential that the mechanisms by which pregabalin induces rewarding effects are further understood.

Glutamate transporters are significantly affected by drug abuse and addiction [14], and glutamate transporter 1 (GLT-1) is accountable for 90% of glutamate uptake [15]. GLT-1 expression is downregulated in individuals with a history of drug abuse and addiction, and this can provoke alcohol, cocaine, and methamphetamine addiction [16,17,18]. Conversely, GLT-1 upregulation attenuates drug-seeking behavior [17]. Drug abuse also downregulates the cystine/glutamate antiporter (xCT), which is responsible for glutamate and cysteine exchange in glial cells. Furthermore, GLT-1 and xCT downregulation increases glutamate concentration, which then causes the overactivation of postsynaptic receptors such as mGlu-1 [19] and mGlu-5 [20,21]. In contrast, blocking these receptors in the nucleus accumbens (NAcc), the reward center of the brain [22], is associated with a reduction in both drug-seeking behavior and addiction relapse [23,24,25,26].

N-acetylcysteine (NAC), an acetylated form of cysteine and a glutathione (GSH) precursor, is a source of scientific enthusiasm due to its efficacy in drug-addiction treatment [27,28]. In humans, NAC is known for its role in mitigating cocaine and nicotine dependence [29,30,31]. This drug was shown to normalize glutamatergic functions and reduce drug-seeking behavior [32,33]. Likewise, NAC was revealed to diminish heroin drug-seeking effects in rats [34]. NAC, therefore, emerged as a potential candidate for the treatment of nicotine, heroin, and cocaine drug-seeking effects [34,35,36]. NAC can upregulate GLT-1 and xCT expression, which in turn regulate glutamate homeostasis by increasing the exchanging amount of glutamate by glial cells [27,37,38]. Therefore, in this study, we assessed the role of NAC on pregabalin induced-seeking behavior using the conditioned place preference (CPP) paradigm.

The CPP paradigm is a model that was extensively used for studying the preference behavior of animals [39,40,41,42]. This model was used to study the effects of several enforcers such as drugs and food. The CPP strategy is characterized by two periods of investigation and two compartments, whereby a reinforcing stimulus during the first period is evaluated during a second (test) period where animals are allowed to choose between the enforcer-paired and nonpaired compartments [41,42,43]. Even though the CPP experiment is a widely used strategy, there are many variables that may affect the results, such as flooring, apparatus design, experimental design, and cues [39,43]. Therefore, in this study, an unbiased CPP where animals showing strong initial preference were excluded from the study to examine the role of NAC on pregabalin induced-seeking like behavior.

## 2. Materials and Methods

### 2.1. Animals

Thirty-six male mice (BALB/c) were purchased from King Fahd Medical Research Center (Jeddah, Saudi Arabia) (weight, 25–30 g) at the beginning of the study. The mice were housed in groups at optimal temperature (21 °C) and humidity (30%) levels, with ad libitum access to water and food; they were allowed to acclimate for 1 week before the experiments. All experiments were conducted during the light cycle of 8:00–10:00 a.m. 

### 2.2. Drugs

Both pregabalin (kindly provided by the research and development department at Jamjoom Pharma, Jeddah, Saudi Arabia) and NAC (Sigma-Aldrich, St. Louis, MO, USA) were reformulated in normal saline solution (0.9% NaCl) before use.

### 2.3. Experimental Design

**Figure 1:**
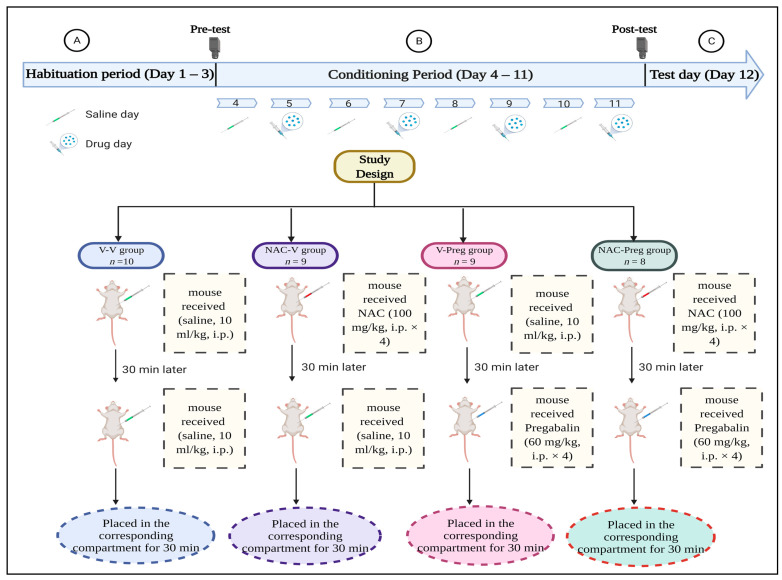
Experimental design to evaluate effect of N-acetylcysteine (NAC) on pregabalin-induced conditioned place preference (CPP). (**A**) Each mouse was permitted to explore both conditioning compartments for 30 min and was tested at Day 3. (**B**) During the conditioning period, each mouse received two injections/day of either saline or NAC (100 mg/kg) in the home cage as a 30 min pretreatment, followed by pregabalin (60 mg/kg) or vehicle. The next day, each mouse received two injections/day of only the vehicle as pre- and post- treatment, and that was repeated for 8 days. (**C**) At Day 12, post-test was conducted.

The experimental scheme is shown in Figure 1. Mice were randomly assigned to 1 of 4 groups treated with different treatment regimens for 8 consecutive days throughout the conditioning period. Mice in Group 1 (V-V group, *n* = 10) were given a vehicle (saline, 10 mL/kg), followed by another injection of the vehicle after 30 min. Group 2 (NAC-V group, *n* = 9) received intraperitoneal (i.p.) injections of NAC (pretreatment, 100 mg/kg, i.p. × 4) followed by vehicle injections 30 min after. Group 3 (V-Preg group, *n* = 9) received vehicle pretreatment 30 min before pregabalin injection (60 mg/kg, i.p. × 4). This dose of pregabalin was selected because it induced place preference in a previous study [13]. Moreover, pregabalin at low doses (30 mg/kg) or less is less effective than high doses are, which were reported in numerous case studies in inducing rewarding effects [44,45,46,47,48]. Lastly, Group 4 (NAC-Preg group, *n* = 8) received NAC pretreatment (100 mg/kg, i.p. × 4) 30 min before pregabalin injection (60 mg/kg, i.p. × 4). Following conditioning, mice were assessed for drug-seeking like behavior.

#### 2.3.1. CPP Model Apparatus

The CPP apparatus was produced from acrylic material. The apparatus contained two conditioning compartments that were identical in size (35 × 35 × 50 cm), and one external start box (10 × 15 × 10 cm) as previously explained [13]. Compartments were marked with distinctive visual and tactile cues. The first compartment had round holes in its flooring and rough white walls lined with horizontal black stripes. The other compartment had flooring with rectangular holes and smooth black walls that were lined with white vertical stripes.

#### 2.3.2. Habituation Period

The habituation period consisted of 3 days. Each mouse was placed in the start box with a closed gate for 3 min. Thereafter, each mouse was permitted to explore both conditioning compartments for 30 min. On Day 3, mice were allowed to freely explore both conditioning compartments. The experiment was recorded with a digital camera, and time spent was calculated using ANY-maze software (Stoelting Co; Wood Dale, IL, USA). The unbiased method for assigning groups was implemented. If a mouse stayed in one compartment for >67% of total test time, that mouse was excluded from the study [49,50,51]. According to this criterion, 4 mice were excluded due to increased initial preference. This step was performed to determine the unbiased preference baseline [52,53]. The mice were then randomly assigned to 1 of the 4 groups.

#### 2.3.3. Conditioning Period

Following the habituation period, each mouse was administered its treatment (i.p. × 4) according to the group assigned throughout the conditioning period (Days 4–11). The mice were administered an injection of either saline or 100 mg/kg NAC 30 min beforehand as pretreatment, and then given either pregabalin or vehicle as posttreatment, placed in one of the closed compartments (pregabalin-paired), and allowed to explore for 30 min. The next day, the mice received vehicle injections as pretreatment and post-treatment and were placed in the other closed conditioning compartment (vehicle-paired) for 30 min. This procedure was repeated for 8 days.

The postconditioning test was conducted on Day 12, when each mouse was allowed to travel freely in both the compartments for 30 min. This experiment was also recorded with a digital camera, and the time each mouse spent in both compartments was measured using ANY-maze software (Stoelting, Wood Dale, IL, USA)

### 2.4. Statistical Analysis

Two-way ANOVA with repeated measures (time × compartment) was used for the evaluation of the time spent in the pregabalin- or saline-paired chamber during the habituation and conditioning periods. This statistical analysis was chosen on the basis of previous studies [39,50,51,54,55,56]. When significant effects were discovered, Tukey’s multiple comparison post hoc test was applied. Data were analyzed using GraphPad Prism (GraphPad Software, San Diego, CA, USA), with the level of significance set at *p* < 0.05.

## 3. Results

**Figure 2:**
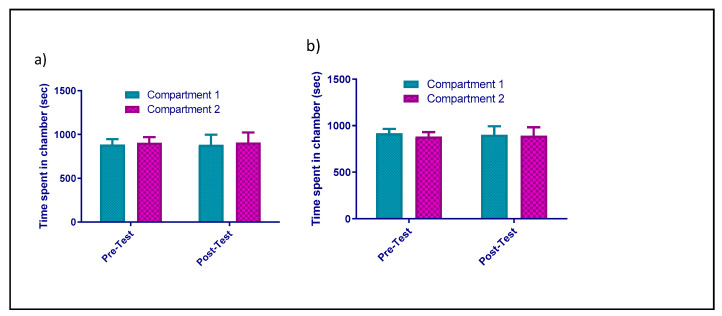
(**a**) Effect of vehicle on CPP. Time spent (mean ± SEM) of vehicle-treated groups was examined. There was no significant difference in time spent in pre- and post-test in control group. (**b**) Effect of NAC on CPP. Time spent (mean ± SEM) did not significantly change in the vehicle- and NAC-treated groups in the pre- and post-test.

**Figure 3:**
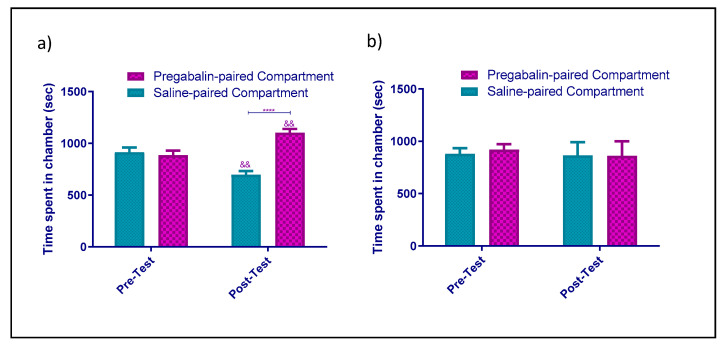
(**a**) Effect of pregabalin (60 mg/kg) on CPP. Time spent in pregabalin-paired compartment was significantly higher than that spent in the vehicle-paired compartment in the post-test. Time spent was significantly increased in the pregabalin-paired compartment post-test when compared to the saline and pregabalin-paired compartment pretest. (**b**) Effect of NAC-pretreatment on pregabalin-induced CPP. Time spent (mean ± SEM) was not significantly changed in pregabalin-paired compartment as compared to the vehicle-paired compartment pre- and post-test. ****: *p* < 0.0001; (&&: *p* < 0.01).

In Group 1, which was the control group where animals received saline, there were no significant differences with regard to time (F_1,9_ = 0.002922, *p* = 0.9581), compartment (F_1,9_ = 0.02993, *p* = 0.8665), or interaction between time and compartment (F_1,9_ = 0.0006267, *p* = 0.9806) during the conditioning phase, as shown in Figure 2a. In Group 2, where the animals received NAC (100 mg/kg), the effect of NAC on CPP was studied. Pretreatment with NAC did not affect CPP. Statistical analyses revealed no significant effects of NAC on time (F_1,8_ = 3.053, *p* = 0.1187), compartment (F_1,8_ = 0.1971, *p* = 0.6688), or interaction between time and compartment (F_1,8_ = 0.008029, *p* = 0.9308) during the conditioning period, as shown in Figure 2b. In Group 3, where the animals received pregabalin (60 mg/kg), the effect of pregabalin on CPP was studied, and pregabalin had induced CPP at a dose of 60 mg. Statistical analysis displayed a significant effect on compartment (F_1,8_ = 6.344, *p* = 0.0359) and significant interaction between time and compartment (F_1,8_ = 51.75, *p* < 0.0001), but there were no significant differences with regard to phases (F_1,8_ = 1.146, *p* = 0.3157). Tukey’s test revealed no significant difference in time spent when comparing the pregabalin- and vehicle-paired compartments in the pretest (*p* = 0.8958; Figure 3a). However, time spent in the pregabalin-paired compartment was significantly higher than that spent in the vehicle-paired compartment in the post-test (*p* < 0.01; Figure 3a). In addition, time spent in the pregabalin-paired compartment was higher in the post-test as compared to in the pretest (*p* = 0.0042; Figure 3a). Lastly, in Group 4, where mice were given NAC pretreatment and were then subjected to pregabalin, no significant changes were revealed in time (F_1,7_ = 1.000, *p* = 0.3506), compartment (F_1,7_ = 0.01108, *p* = 0.9191), or interaction between time and compartment (F_1,7_ = 0.04368, *p* = 0.8404; Figure 3b) in the pre- and post-test.

## 4. Discussion

Pregabalin at low doses (30 mg/kg) or less is less effective than high doses are, which were reported in numerous case studies in inducing rewarding effects [44,45,46,47,48]. Previous reports on animal models revealed that a low dose of pregabalin (up to 30 mg/kg) did not induce the CPP model. In humans, standard doses of pregabalin range from 75 to 600 mg/day [57], and patients who experience euphoria and withdrawal symptoms were reported to abuse pregabalin by taking larger quantities or using it for a longer time than that prescribed [9,10,11,12]. Consistent with this, one study found that the CPP in a mouse model was induced using higher doses of pregabalin (60 and 90 mg/kg) [13]. The consistency of the results supported the validity of using these doses in animal experiments to test the reward-seeking properties of pregabalin. In this study, as expected, a 60 mg/kg dose of pregabalin induced drug-seeking behavior in a CPP mouse model of drug addiction (Figure 3a).

Pregabalin decreases the release of glutamate and reduces the activation of the AMPA receptor [58,59,60]. In a tooth-pain model, the release of glutamate, which was induced by mustard oil, was blocked by the systemic administration of pregabalin [61]. Furthermore, another study demonstrated that the release of glutamate was decreased in the medullary dorsal horn by the administration of pregabalin in a rodent model [62]. Other evidence from rodent model studies showed that pregabalin reduces neuropathic pain by inhibiting the release of glutamate in the spinal cord [63]. A 2013 study showed that the reduction in glutamate and glutamine release was attributed to pain reduction, and a reduction in brain connectivity was observed using neuroimaging [64]. Another mechanism that involves the augmentation of glutamate transporter activity [65] and a reduction in neuronal plasticity was suggested for pregabalin analgesic properties [66]. In contrast to our study, these experiments used a low dose of pregabalin (10–30 mg/kg) or a neuropathic pain model. In our study, a high dose of pregabalin was found to induce CPP, and it was speculated that the concentration of glutamate had probably increased. Therefore, another study is warranted to specifically determine the effects of high doses of pregabalin on the release of the GLT-1 receptor and glutamate.

The mechanism by which pregabalin induces drug-seeking like behavior is poorly understood. However, the glutamatergic system was implicated in drug-seeking behavior [17,19]. Glial excitatory amino acid transporter GLT-1 is the primary regulator of glutamatergic homeostasis in the brain [15], and many studies linked glutamatergic imbalance in the NAcc along with GLT-1 downregulation to drug-seeking behavior [67,68,69]. For instance, the downregulation of GLT-1 expression is associated with cocaine-seeking behavior [70]. Moreover, this glutamatergic-system disturbance is associated with glutamate spillover as glutamate uptake from the synapse decreases [71]. Furthermore, elevated glutamate concentration at the synapses leads to the potentiation and enhancement of many receptors (e.g., mGlu 5 and NMDA) that increase drug-seeking behavior in mice [71]. Pregabalin (60 mg/kg)-induced CPP might occur in part due to the downregulation of xCT and GLT-1 expression, which increases glutamate concentration at the synapses. One limitation of this study is that GLT-1 and xCT expression levels were not measured; therefore, additional studies are warranted to investigate molecular changes in GLT-1 and xCT in pregabalin-induced drug-seeking-like effects.

Another possible mechanism of pregabalin-induced seeking behavior might be due to its effects on mGlu2/3 receptors, which are presynaptically expressed. These presynaptic receptors are involved in reward- and drug-seeking behavior by controlling the release of glutamate in the NAcc and prefrontal cortex [72]. The activation of the mGlu2/3 receptor reverses drug-seeking behavior by blocking the release of glutamate [71], whereas antagonists of these receptors increase the release of glutamate in the NAcc [73]. Pregabalin may work by causing an initial decrease in the release of glutamate, which may then lead to the desensitization or downregulation of these presynaptic mGlu2/3 receptors [73]. These neuroadaptations and impaired mGlu2/3 functions were reported following repeated exposure to several drugs of abuse [74]. Moreover, in our current study, NAC blocked the pregabalin-induced CPP, which may have been due to its improvement of glutamatergic tone on the presynaptic mGlu2/3 receptors [33,75]. Additional studies are warranted to study the molecular changes of mGlu2/3 in response to the pregabalin-induced CPP.

NAC is an effective treatment, especially in cocaine-free patients [29,30]. Furthermore, prolonged treatment with NAC restores the expression levels of GLT-1 and xCT, and reduces drug-seeking behavior [32,33]. Along with its potentially therapeutic effects regarding drug abuse, NAC may be used to treat different neurological diseases, such as Alzheimer’s disease and bipolar disorder, which may involve the glutamatergic system [76]. For instance, pretreatment with NAC in rats with ischemic stroke can restore GLT-1 expression; hence, NAC treatment may prevent the glutamate-induced neurotoxicity observed in these neurological diseases [77]. Taken together, these studies suggest that NAC can reverse the neurophysiological changes induced by drug abuse [28,78]. Additionally, drug-abuse treatment with NAC is more promising than other treatments are due to its unique therapeutic potential and relative safety. Moreover, pretreatment with NAC (100 mg/kg) is effective in preventing pregabalin-induced seeking.

Alternate mechanisms associated with the therapeutic potential of NAC in neurological disorders need to be elucidated [77] and may involve its antioxidant properties [40,79,80]. GSH engages in the enzymatic and nonenzymatic detoxification of reactive oxygen species (ROS) [81]. Additionally, NAC, which is a GSH precursor, scavenges ROS in neurons [82] and stabilizes the oxidative status by countering oxidative-stress markers (e.g., nitric oxide and superoxide radicals) [83,84]. Many studies also connected glutamate neurotoxicity in drug abuse with increased oxidative stress [85], and NAC could improve the oxidative-stress profile of rats exposed to alcohol [49,86,87]. Additional studies are warranted to further study the role of NAC on oxidative-stress markers in pregabalin-induced CPP.

Another limitation of this study is that the treatment group (NAC-Preg) was not directly compared with the pregabalin group. However, the group was treated with NAC did not show a preference for the drug-paired compartment, which can be attributed to the blocking effect of NAC on pregabalin. On the other hand, a strength of this study was the use of the unbiased CPP paradigm, which required that each animal spend less than 67% of total time in one compartment during the pretest period. Therefore, mice were excluded if they spent more than 67% of the total time in one compartment. The unbiased paradigm was used to avoid any impact of the CPP flooring or walls and improve the accurate assessment of conditioning cues. Another strength of our study is that two-way ANOVA with repeated measures was utilized for CPP data. Different within-group variables can affect CPP [41,42,88]. Cunningham et al. explored this issue and found a significant interaction between groups and other variables that could alter the interpretation of CPP data [41,42,88]. Therefore, a comparison between dependent variables was used to ensure the credibility of the results.

## 5. Conclusions

In conclusion, this study revealed for the first time that the administration of NAC reduced pregabalin-seeking behavior. The glutamatergic system was also proposed to be involved in this process. Further studies are warranted to confirm whether NAC-induced changes in GLT-1 or xCT expression levels represent contributing factors to the observed effects of NAC.

## Data Availability

Available upon reasonable request.
